# Underwater Optical Wireless Communications: Overview

**DOI:** 10.3390/s20082261

**Published:** 2020-04-16

**Authors:** Giuseppe Schirripa Spagnolo, Lorenzo Cozzella, Fabio Leccese

**Affiliations:** 1 Dipartimento di Matematica e Fisica, Università degli Studi “Roma Tre”, 00146 Roma, Italy; schirrip@uniroma3.it (G.S.S.); lorenzo.cozzella@uniroma3.it (L.C.); 2 Dipartimento di Scienze, Università degli Studi “Roma Tre”, 00146 Roma, Italy

**Keywords:** underwater optical wireless communication (UOWC), underwater communication, visible-light communications, ocean optics

## Abstract

Underwater Optical Wireless Communication (UOWC) is not a new idea, but it has recently attracted renewed interest since seawater presents a reduced absorption window for blue-green light. Due to its higher bandwidth, underwater optical wireless communications can support higher data rates at low latency levels compared to acoustic and RF counterparts. The paper is aimed at those who want to undertake studies on UOWC. It offers an overview on the current technologies and those potentially available soon. Particular attention has been given to offering a recent bibliography, especially on the use of single-photon receivers.

## 1. Introduction

In order to give a basic overview for the purpose of the special issue “Underwater wireless optical communications”, we will provide a short summary to highlight the perspectives of UOWC technologies. Without pretending to be exhaustive, in this work, the main points to which research attention should be directed will be indicated.

Currently the use of wireless communications is very common in a wide range of terrestrial devices. In the underwater world, the application of wireless communications is of great interest to the military, industry, and the scientific community [1,2]. Acoustic systems have enjoyed great success under water owing to their ability to communicate over many kilometers pushing the research in this field with the aim to further improve this technology. Extensive studies are conducted to improve the performance of the acoustic communication channels [3,4,5,6,7]. Nevertheless, its performance is linked to the physical nature that limits the bandwidth, causes high latency, produces high transmission losses, time varying multi-path propagation and Doppler’s spread [8,9,10,11,12,13]. These limitations do not allow autonomous underwater vehicles (AUV) to transmit real-time video in high definition via acoustic communication. Therefore, complementary technology is needed that can achieve broadband underwater communications, indeed, real-time video transmissions, including the tele-operation of underwater vehicles and remote monitoring of underwater stations, are becoming an important asset for underwater applications [14,15,16,17]. The RF waves, for their nature, are the more common and diffused technic used on terrestrial communications, but even them are not suitable underwater because, they are strongly attenuated [18]. Additionally, standard acoustic underwater communication, due to its bad performance features such as high bit error rates, large and variable propagation delays and low bandwidth, are particularly vulnerable to malicious attacks [19].

Visible-light communication (VLC) is a technology that can solve these problems. In VLC systems the visible light spectrum (400–700 nm) used for illumination is modulated to transmit data [20,21,22,23,24,25,26]. Similar to VLC systems are underwater optical wireless communication (UOWC), systems where potential light sources are LDs instead of LEDs. Both are extremely interesting—LDs for their feature higher modulation bandwidth respect to LEDs—while the latter, due to their higher power efficiency, lower cost and longer lifetime, seem more suitable for medium bit rate applications.

Table 1 shows the performance features (benefits, limitations and requirements) of the three principal underwater communication technologies: acoustic, radio frequency and optical [27].

Unfortunately, the performance of UOWC is currently limited to short range [28]. Therefore, Therefore, even if submarine optical communication systems are beginning to be commercially available [29], extensive research is being carried out on methodologies and systems for the transfer of broadband optical signals at higher distances.

In the future many underwater applications will use optical communication. However, UOWC technologies can never totally replace acoustic communication. For this reason, studies and researcher on hybrid acoustic/optic communications are carried out [30,31,32,33]. These studies are very promising and should be investigated further. 

**Figure 1:**
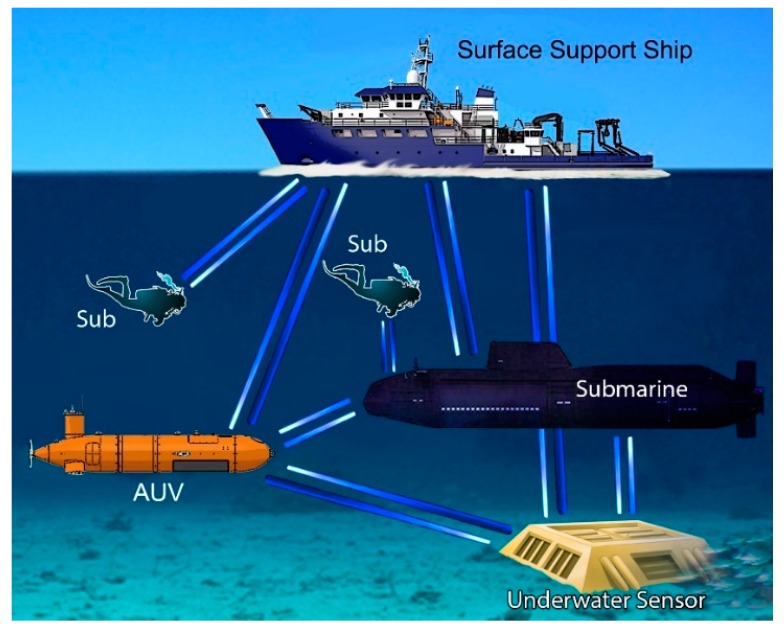
Typical application scenarios of UWOC.

Figure 1 illustrates a generic UOWC scenario. It shows several platforms (divers, ships, submarines, submarine sensors, etc.) connected by beams of light.

## 2. Optical Transmission in the Aquatic Medium

UWOC provides many technical benefits such as e.g., high rates of data transmission, secure links, but also economical ones, such as low installation and operational costs. Moreover, since the optical band is not included in the telecommunications regulations, it does not require payment of licensing fees and tariffs [34,35,36,37,38,39,40].

The main disadvantage of underwater optical communication is that the water is a medium that highly absorbs optical signals; the second problem is optical scattering due to the particles present in the sea. Anyway, with respect to the visible spectrum, seawater has a lower absorption in the blue/green zone. Exploiting this physical feature, working with signals with wavelengths belonging to the blue/green region of the spectrum, high speed connections can be attained according to the type of water. Lowest attenuation is centered at 460 nm in clear waters, but this wavelength shifts to higher values in dirty waters, reaching values around to 540 nm, e.g., for coastal waters [41,42,43,44,45,46].

The bulk optical properties of water can be divided into two mutually exclusive groups: inherent and apparent [47,48,49]. Inherent properties describe those optical parameters that depend only on the medium and from the composition of the medium and from the particulate substances existing inside it. Instead, apparent properties are not dependent only by the medium, but are linked to the geometric structure of the illumination including therefore directional properties.

Absorption and scattering are two phenomena impairing the propagation of light in water. These effects lead to loss and deviation of light photons, respectively. Generally, to describe the propagation in water of collimated light in low scattering regimes, the spectral beam attenuation coefficient c(λ) is used; this parameter is wavelength function. This coefficient is a sum of the effects of the absorption coefficient and of the scattering one, respectively called a(λ) and b(λ), and so defined as [50,51,52]:

(1) c(λ)=a(λ)+b(λ)

The absorption and scattering coefficients, with inverse meter units, are determined by the contribution of water molecules, particulate algal/sediment matters, and colored organic contents dissolved [53,54,55].

**Figure 2:**
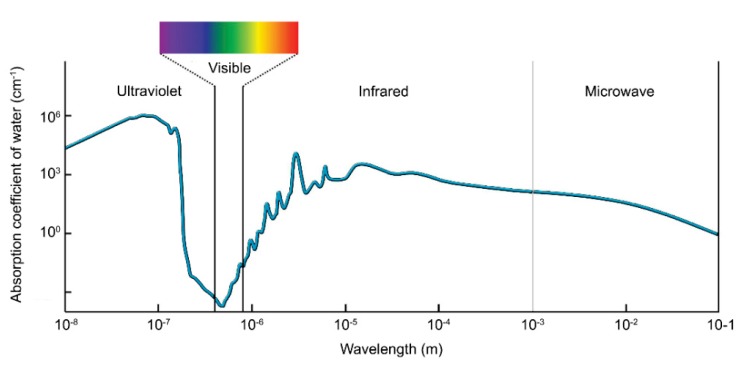
Absorption coefficient of pure seawater for different transmission wavelengths.

The water absorption coefficient was exhaustively measured with high accuracy from 300 nm to 700 nm, showing a minimum between 400 nm–500 nm [56,57,58,59,60]. Figure 2 illustrates the absorption coefficient of light in pure seawater.

Obviously, in addition to wavelength and type of particles in solution/suspension, the level of turbidity, largely affect both absorption and scattering [61]. Among the possible particles detectable in seawater, the organic ones and phytoplankton are particularly important for the optical properties of seawaters. In fact, the chlorophyll pigments of the phytoplankton present the property of strongly absorbing light in the blue and red spectral regions. These particles condition the seawater absorbance contributing to the formation of the scattering coefficient value [62].

Generally, both absorption and scattering limit the link distance of a UWOC system. The scattering leads a reduction in the number of photons collected by the receiver. Furthermore, in a turbid underwater environment, several photons may arrive at the receiver with delays and cause intersymbol interference (ISI) effects [63].

The values of a(λ) and b(λ), in addition to the wavelength, vary with the water type. Usually, for simplicity, but without loss of generality, different values of chlorophyll concentration C are used to characterize the different type of waters [47,62,64,65]. In this way, the absorption coefficient a(λ) and the scattering coefficient b(λ) can be expressed as a function of the wavelength λ and of the concentration C [66]:

(2) $$a(\lambda )=\left[a_{w}(\lambda )+0.06a_{c}(\lambda )C^{0.65}\right]\left\{1+0.2\exp\left[-0.014\left(\lambda -440\right)\right]\right\}$$

(3) $$b(\lambda )=0.30\frac{550}{\lambda }C^{0,62}$$

where $a_{w}$ points out the pure water absorption coefficient while, $a_{c}$ is a nondimensional number, statistically derived that points out the absorption coefficient specific for the chlorophyll. Therefore, the chlorophyll concentration C, expressed in mg·m^−3^, can be used as the free parameter to calculate a(λ) and b(λ).

The measured values for the absorption a(λ), for the total scattering b(λ) and for the extinction c(λ) are outlined in Table 2 [19,43,56]; usually four major seawater types are considered. It is important to note that the absorption measurements have been obtained in a spectral band with λ centered at 532 nm.

Beer’s law is commonly used to describe the propagation loss factor ($L_{P}$) as a function of wavelength (λ) and distance (z). The propagation loss factor [67,68,69,70]:

(4) $$L_{P}(\lambda ,\;z)=h\;\cdot \;\exp\left[-c\left(\lambda \right)\cdot z\right]$$

In Equation (4), c(λ) represents the cumulative attenuation coefficient as defined in Equation (1), while h is a constant. Unfortunately, the Beer’s Law disregards the indirect paths and, obviously, with the increase of the distance, the multiple scattering conditions the channel losses while, some photons that run through these non-line of sight paths may arrive to the receiver causing errors of interpretation. Respect to the Beer’s Law model, a function with two exponentials more accurately approximates the power loss for long distance underwater channel. A first exponential considers the attenuation loss length less than the diffusion length and another one greater than the diffusion length. Therefore, Equation (4) can be rewritten as [71]:

(5) $$L_{P}(\lambda ,\;z)=h_{1}\;\cdot \;\exp\left[-c_{1}\left(\lambda \right)\cdot z\right]+h_{2}\;\cdot \;\exp\left[-c_{2}\left(\lambda \right)\cdot z\right]$$

Currently, a theoretical model capable of describing long distance optical communication in a practical underwater environment is not available.

In ocean water, the performance of an underwater optical communication system is mainly limited by oceanic turbulence, which is defined as the fluctuations in the index of refraction resulting from temperature and salinity variations. By means of the Monte Carlo method, it is possible to implement a complete “model” to evaluate also the propagation through weak oceanic turbulence [72,73,74,75,76,77,78,79,80,81]. In any case, the characterization of the transmission channel model is a critical point for the development of the UWOC systems. Therefore, continuous studies and research are necessary to obtain models that always better adapt to real conditions. In Equation (5), the parameters $h_{1}$, $c_{1}$, $h_{2}$, and $\;c_{2}$ can be calculated by the least mean square fitting algorithm; example of computed parameters by means of Monte Carlo simulation is shown in Table 3 [82,83].

**Figure 3:**
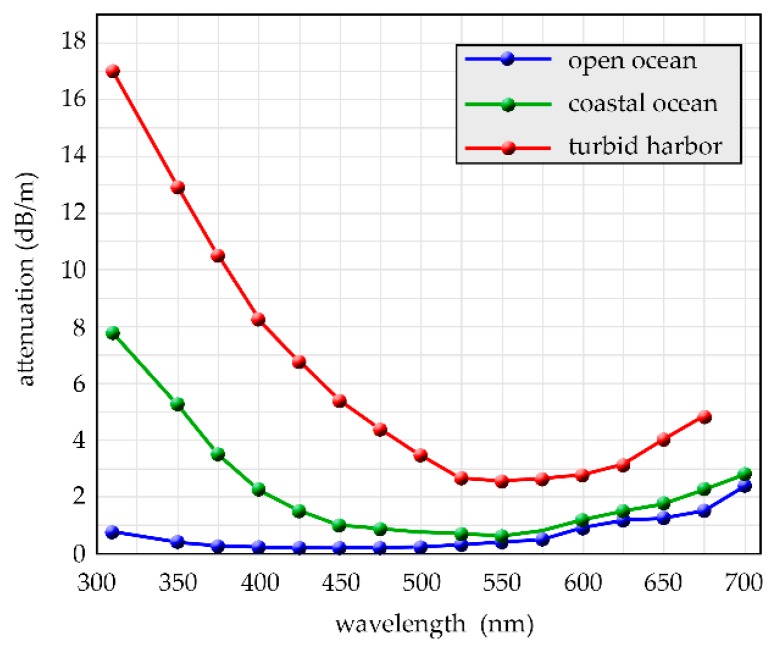
Attenuation in dB/m for different ocean waters.

As said, underwater the light shows less attenuation in the blue/green wavelength range. However, although light attenuation in seawater is minimum in the blue-green region, the optimal wavelength for underwater optical link is conditioned from the inherent optical properties of the water, which can largely vary in different geographic places. Figure 3 shows typical attenuation (dB/m) versus wavelength for various ocean waters [50,84]. 

## 3. Basic Components of Underwater Optical Wireless Communications (UOWCs)

**Figure 4:**
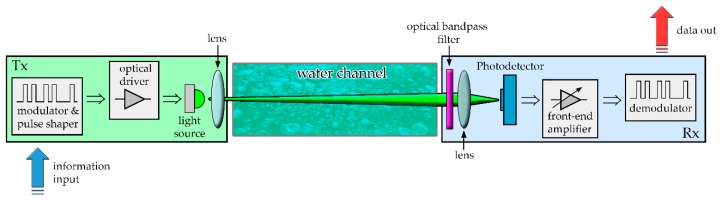
Schematic of a typical UOWC link. The transmitter (TX) is composed of a modulator, optical driver, light source and projection lens. The receiver (RX) is made of optical bandpass filter, photodetector, Low noise electronics and demodulator.

A UOWC link can be schematized in three parts, the transmitter unit, the water channel and the receiver module. The schematic in Figure 4 shows the components of a typical system.

### 3.1. The Transmitter (TX)

The transmitter consists of four principal components: a modulator and pulse shape circuit, a driver circuit, that converts the electrical signal to an optical signal suitable for transmission and a lens to realize the optical link configuration.

The modulator and pulse shape are critical points of the system. Recent UOWC studies have tried to characterize the performance of communication systems using different modulation techniques in order to increase together the data transmission rate and the link distance [85,86,87].

Typical RF modulating schemes are not applicable in VLC. The three modulation schemes standardized by IEEE [88,89,90,91] are OOK, IM-DD and CSK. Among them, the easiest applicable to the UOWC schema is the non-return zero with OOK (NRZ OOK), which is binary code where “1” is represented by a light pulse while “zero” means no pulses. IM-DD is a transmission scheme in which the intensity of the optical source is modulated by the signal, and the demodulation is achieved through direct detection of the optical carrier and conversion using a photo-detector. Color shift keying (CSK) is a visible light communication (VLC) modulation scheme, designed for multi-color light emitting diodes (LEDs), so it is not applicable in a UOWC.

In addition to the previous ones, orthogonal frequency division multiplexing (OFDM) is used in multiple sub-carrier modulation (MSM) techniques. MSM techniques are applicable for scenarios where single transmitter provides homogenous transmission of data to several receivers. In MSM, OFDM symbols are modulated onto individual sub-carriers which combine to modulate onto instantaneous power of the transmitter due to orthogonality of sub-carriers. Different OFDM schemas exist for UOWC, such as QPSK and QAM [92,93].

For UOWC systems, the function of the transmitter is to transform the electrical signal in optical one, projecting the carefully aimed light pulses into the water. The optical light sources are based on LED or LD one [94,95,96,97,98,99,100,101,102]. 

**Figure 5:**
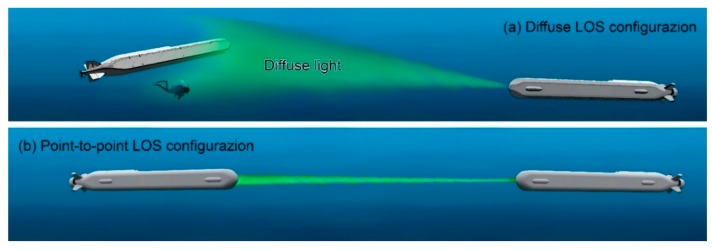
Examples of different underwater optical wireless link configurations.

In underwater optical communication, the connection between transmitter and receiver can be of two main types (see Figure 5) [20,51]: (a) diffuse line-of-sight (diffuse LOS) configuration; (b) point-to-point line-of-sight (LOS) configuration.

Diffused LOS configurations use diffused light sources, such as high-power, highly efficient LEDs. They present large divergence angles to permit broadcasting UOWC from one node (the transmitter) to more receiver nodes as shown in Figure 5a. This configuration, due to the wide interaction volume light-water, is very sensitive to the attenuation caused by the water. This leads to relatively short communication distance and low transmission data rate. These are the two more important disadvantageous of this configuration.

The point-to-point LOS configuration well shown in Figure 5b is the more common link configuration used in UOWCs [103,104]. In this arrangement, the receiver is placed in such a way to detect the light beam directly aimed in the direction fixed by the transmitter. Obviously, since these systems commonly use light sources with narrow divergence angles, typically lasers, they require precise pointing between TX and RX. This constraint can strongly limit the performance of UOWC systems in case of turbulent water environments and can cause heavy problems when the transmitter and the receiver are non-stationary nodes; this case is particularly felt for AUVs and remotely operated vehicles (ROVs) [105,106].

The diffused LOS configuration is well suited for short-range transmission between moving objects. On the other hand, the point-to-point LOS configuration is preferred over long distances and when precise aiming between TX and RX is possible.

In order to implement these link configurations, it is necessary to use suitable projection system that employ lens groups. In order to expand the UOWC covered area and improve the system performance, various transmission schemes can be used [107,108,109,110,111,112,113].

Recent studies are aimed at developing systems where a narrow-beam from an underwater vehicle is “exactly” pointed towards the receiving terminal of a second underwater vehicle. In these systems, the transmitting module implements a scan function that allows the communication channel to remain active even with TX and RX in motion [114,115]. 

### 3.2. The Receiver (RX)

In many applications it is important to select a specific wavelength that impacts on the light detector [116]. The light reaching the receiver should have no noise introduced by sunlight and avoid the presence of other light sources [117]. To try to solve this problem, the wavelength band (the one transmitted) is selected by using a narrow optical band-pass filter [118].

When the receiver receives the transmitted optical signal, it transforms it into an electric signal by using photodetectors. Many different types of photodetectors are currently commonly used, e.g., photodiodes. These devices, for their characteristics of small size, suitable material, high sensitivity and fast response time, are commonly used in optical communication applications. There are two types of photodiodes: the PIN photodiode and the avalanche photodiode (APD). 

Unfortunately, due to the high detection threshold and high noise intensity, linked to the trans-conductance amplifier, that limit their practical application, photodiodes are not advisable for long distance UOWC systems. For traditional detection devices and methods, due to the exponential attenuation of the water, the optical communication distance is less than 100 m [56,70].

Recent studies are focused on the possible application of single photon avalanche diodes (SPADs) technology to UOWC systems. Avalanche photodiodes have a similar structure as PIN ones but operate at a much higher reversed bias. This physical characteristic allows a single photon to produce a significant avalanche of electrons. This mode of operation is called the single-photon avalanche mode or Geiger’s mode [87,88]. The great advantage of SPADs is that their detectors do not need to a trans-conductance amplifier. This intrinsically leads to the fact that optical communications implemented with this kind of diodes can provide high detection, high accuracy and low noise measurements [119,120,121,122,123,124,125,126,127,128,129,130,131,132]. RX sensors based on SPADs still require further in-depth studies. For these systems, it is important to check the immunity to external disturbances and develop specific modulation schemes.

## 4. Conclusions

Underwater Optical Wireless Communication (UOWC) has recently emerged as a unique technology facilitating high data rates and moderate distance communication in undersea environments. Many applications with large amount of data such as real-time video transmission and control of remotely operated vehicles could greatly benefit from UOWC. Nowadays, UOWC systems usable under real operating conditions are rarely available, therefore a lot of research in this area has yet to be done.

## Figures and Tables

**Table 1:** Comparison of underwater wireless communication technologies.

Parameter	Acoustic	RF	Optical
Attenuation	Distance and frequency dependent (0.1–4 dB/km)	Frequency and conductivity dependent (3.5–5 dB/m)	0.39 dB/m (ocean) 11 dB/m (turbid)
Speed	1500 ms^−1^	2.3 × 10^8^ ms^−1^	2.3 × 10^8^ ms^−1^
Data Rate	kbps	Mbps	Gbps
Latency	High	Moderate	Low
Distance	more than 100 km	≤10 m	10–150 m (500 m potential)
Bandwidth	1 kHz–100 kHz	MHz	150 MHz
Frequency Band	10–15 kHz	30–300 MHz	5 × 10^14^ Hz
Transmission Power	10 W	mW–W	mW–W

**Table 2:** Typical values of a(λ), b(λ)  and c(λ) for different water Type; work out with λ=532 nm. *Pure sea waters*: absorption is the main limiting factor. *Clear ocean waters*: they have a higher concentration of dissolved particles that affect scattering. *Coastal ocean waters*: they have a much higher concentration of planktonic matters, detritus, and mineral components that affect absorption and scattering. *Turbid harbor waters*: they have a very high concentration of dissolved and in-suspension matters.

Water Types	*C* (mg/m^3^)	$a\left(\lambda \right)\;(m^{-1})$	$b\left(\lambda \right)\;(m^{-1})$	$c\left(\lambda \right)\;(m^{-1})$
Pure sea water	0.005	0.053	0.003	0.056
Clear ocean water	0.31	0.069	0.08	0.151
Costal ocean water	0.83	0.088	0.216	0.305
Turbid harbor water	5.9	0.295	1.875	2.170

**Table 3:** Example of Optical Parameters for Different Types of Water; work out with λ=532 nm.

Wavelength (nm)	*C* (mg/m^3^)	$h_{1}\left(\lambda \right)\;(m^{-1})$	$c_{1}\left(\lambda \right)\;(m^{-1})$	$h_{2}\left(\lambda \right)\;(m^{-1})$	$c_{2}\left(\lambda \right)\;(m^{-1})$
Pure sea water	0.005	0.2000	0.0657	0.0046	0.2634
Clear ocean water	0.31	0.1000	0.1508	0.1589	0.4937
